# Diagnostic Leukapheresis Enables Reliable Transcriptomic Profiling of Single Circulating Tumor Cells to Characterize Inter-Cellular Heterogeneity in Terms of Endocrine Resistance

**DOI:** 10.3390/cancers11070903

**Published:** 2019-06-28

**Authors:** Florian Reinhardt, André Franken, Franziska Meier-Stiegen, Christiane Driemel, Nikolas H. Stoecklein, Johannes C. Fischer, Dieter Niederacher, Eugen Ruckhaeberle, Tanja Fehm, Hans Neubauer

**Affiliations:** 1 Department of Obstetrics and Gynecology, University Hospital and Medical Faculty of the Heinrich-Heine University Duesseldorf, 40225 Duesseldorf, Germany; 2 Department of General, Visceral and Pediatric Surgery, University Hospital and Medical Faculty of the Heinrich-Heine University Duesseldorf, 40225 Duesseldorf, Germany; 3 Institute for Transplantation Diagnostics and Cell Therapeutics, University Hospital and Medical Faculty of the Heinrich-Heine University Duesseldorf, 40225 Duesseldorf, Germany

**Keywords:** breast cancer, liquid biopsy, diagnostic leukapheresis, DLA, circulating tumor cells, CTC, single cell profiling, transcriptomic profiling, RT-qPCR

## Abstract

Circulating tumor cells (CTCs) hold great promise with regard to prognosis, treatment optimization, and monitoring of breast cancer patients. Single CTC transcriptome profiling might help reveal valuable information concerning intra-patient heterogeneity relevant to therapeutic interventions. In this study, we combined Diagnostic Leukapheresis (DLA), which is a microfluidic enrichment using the Parsortix^TM^ system, micromanipulation with CellCelector^TM^ and subsequent single cell multi-marker transcriptome profiling. First, a PCR panel consisting of 30 different endocrine resistance and phenotypic marker genes was validated for single cell profiling by using different breast cancer cell lines. Second, this panel was applied to characterize uncultured and cultured CTCs, which were enriched from a cryopreserved DLA product obtained from a patient suffering from metastatic breast cancer resistant to endocrine therapy. Gene expression profiles of both CTC populations uncovered inter CTC heterogeneity for transcripts, which are associated with response or resistance to endocrine therapy (e.g., *ESR1, HER2, FGFR1*). Hierarchical clustering revealed CTC subpopulations with different expressions of transcripts regarding the CTCs’ differential phenotypes (*EpCAM, CD44, CD24, MYC, MUC1*) and of transcripts involved in endocrine signaling pathways (*FOXO, PTEN*). Moreover, ER-positive CTCs exhibited significant higher expression of Cyclin D1, which might be relevant for CDK4/6 inhibitor therapies. Overall, gene expression profiles of uncultured and cultured CTCs resulted in a partly combined grouping. Our findings demonstrate that multi-marker RNA profiling of enriched single uncultured CTCs and cultured CTCs form cryopreserved DLA samples may provide important insights into intra-patient heterogeneity relevant for targeted therapies and therapy resistance.

## 1. Introduction

Approximately 70% of all breast cancers (BCs) express hormone receptors and are, therefore, endocrine sensitive [1,2]. However, the effectiveness of endocrine therapies (ET) is limited due to primary and secondary endocrine resistance. Several molecular resistance mechanisms were proposed, which include alterations in the estrogen receptor (ER) expression, *ESR1* mutations, altered expression of growth factor receptors, the activation of the PI3K/Akt/mTOR pathway, dysregulation of ER co-activators, altered expression of cell cycle regulators, autophagy, epithelial to mesenchymal transition, and increased tumor heterogeneity [3,4].

Primary tumors consist of several tumor cell subclones, which could lead to therapy resistance and harbor different tendencies to metastasize. BC patients show an early hematogenous dissemination of tumor cells in the course of disease. Circulating tumor cells (CTCs) represent precursor cells of metastatic disease and have become a surrogate marker for prognosis of BC patients [5]. In addition to the prognostic value of CTC counts, their molecular characterization by transcriptomic analysis could reveal valuable information regarding the expression of therapeutic target molecules as well as about possible resistance mechanisms. 

However, the utility of CTCs as “liquid biopsies” in BC is currently limited and challenged by their low frequency in blood [6], which is why intra-tumoral and intertumoral heterogeneity of CTCs cannot be fully addressed. This major challenge can be partly solved by the implementation of diagnostic leukapheresis (DLA) into the CTC enrichment workflow. This method was recently validated in BC patients, where it demonstrated to have no side effects on the patients and their treatment regimen [7,8,9,10]. DLA is able to provide many more CTCs per patient than a normal blood draw which enables in-depth analysis of patient-matched cells in order to get insights into the CTCs’ biology on a single cellular level. These significantly higher numbers of CTCs can be used for various downstream analyses such as the CTC culture [10] and enables isolation of many single CTCs for subsequent parallelized multi-marker analyses, which are technically highly challenging but will also be the key to obtain the information needed to get insights into intra-patient tumor cell heterogeneity. 

In order to use DLA products for transcriptome profiling, the primary aim of this study was to set up a robust, rapid, and cost-efficient workflow for enrichment of single CTCs combining DLA, the microfluidic Parsortix^TM^ system (Angle plc, Guildford, UK) was, and the micromanipulator CellCelector^TM^ (ALS, Jena, Germany) was with subsequent CTC transcriptomic characterization on single cell level. By applying this workflow, we characterized the inter-cellular heterogeneity of single CTCs in terms of possible endocrine resistance mechanisms as well as relevant targets for ET in an endocrine resistant metastasized BC patient. We also compared the first-time single gene expression profiles of uncultured and cultured CTCs (cCTCs) of the same metastatic BC patient. Our data suggest a high plasticity as well as intra-individual heterogeneity of CTCs regarding the expression of endocrine and phenotypic markers. They discriminate different CTC subgroups relevant for ET response and resistance and demonstrate a concurrence of ET relevant markers in cultured and uncultured CTCs. Our findings suggest that DLA and single cell phenotyping of uncultured and cultured CTCs is a practical approach for the exploration of tumor heterogeneity and might have great potential for molecular guided cancer therapy.

## 2. Results

### 2.1. Validation of Single Cell Multi-Marker RT-qPCR Analysis

**Figure 1:**
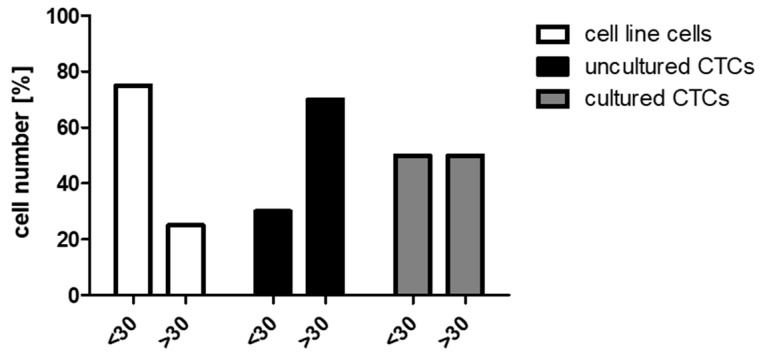
RNA quality of isolated single tumor cells. Signal intensities (Cq < 30: single cells with a Cq < 30 of *ACTB* and a Cq < 30 of *KR19*, Cq > 30: single cells with a Cq > 30 of *ACTB* and/or *KR19*) of single cell line cells (*n* = 8), uncultured CTCs (*n* = 23), and cultured CTCs (*n* = 10). CTCs: Circulating tumor cells

To test whether single cell analysis produces consistent RNA profiles, the expression levels of the reference genes *ACTB*, *UBC*, and *GAPDH* were determined in a cell titration experiment with 10 cells, five cells, and one cell. For all three transcripts, the measured Cq values correlated linearly with the cell numbers (Figure S1). Compared to *UBC* and *GAPDH*, *ACTB* demonstrated the lowest measurable Cq values with all cell numbers. Therefore, expression of the reference gene *ACTB* was selected as the single cell RNA quality marker before in-depth multi-marker analysis. Moreover, previous studies identified *KR19* expression as a marker for identifying CTCs in cancer patients as well as a quality marker for RT-qPCR analysis of CTCs [11,12,13,14]. Based on these reports, we also included *KR19* expression besides *ACTB* expression in addition to an intact cell morphology to select both, best-quality single cells and cDNA-products. Based on Cq values of *KR19* and *ACTB* for single MCF-7 and MDA-MB-231 cells, we defined a Cq < 30 for *ACTB* and *KR19* after pre-amplification as threshold assuming that the total mRNA extracted from such cells is less likely to be degraded. By applying these criteria, 75% of analyzed single cells of a cell line reached a Cq < 30 of *ACTB* and *KR19* suggesting good RNA quality and, thus, it was qualified for further multi-marker analysis (Figure 1). For CTCs, which were selected by intact morphology (bright field microscopy), a nucleus larger than 4 µm in diameter, a positive signal for EpCAM, and a negative signal for CD45, 30% of analyzed CTCs revealed a Cq < 30 of *ACTB* and *KR19* after enrichment via DLA and the Parsortix^TM^ system. For single CTCs, which were cultured of the same cryopreserved DLA product (cCTCs) after enrichment and isolation via the Parsortix^TM^ and CellCelector^TM^ systems, 50% of the cCTCs reached a Cq < 30 of ACTB and KR19 indicating good RNA quality.

**Figure 2:**
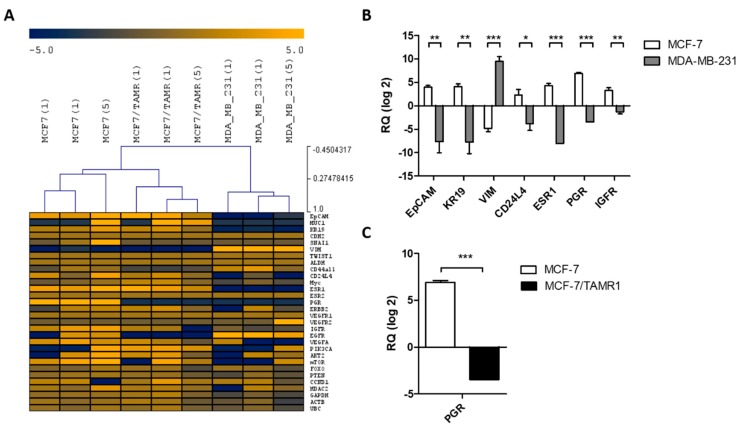
Gene expression of single cells and pools of isolated cell line cells. (**A**) Dendrogram and heat map analyses of Cq measurements of single MCF-7, MCF-7/TAMR1, and MDA-MB-231 cells (*n* = 2 per cell line) and pools of five cells (*n* = 1). Each gene is measured in triplicate. Data are mean centered, with mean expression responses to zero. Yellow indicates high expression and blue color represents downregulation relative to the mean of the pool. (**B**) Bar graph showing significant differences in transcript expression between MCF-7 and MDA-MB-231 cells (*, *p* < 0.05, ** *p* < 0.01, *** *p* < 0.001). (**C**) Bar graph showing significant differences in transcript expression between MCF-7 and MCF-7/TAMR1 cells (*** *p* < 0.001).

We next selected a multi-marker panel of 30 genes from the published literature, which characterizes (a) target genes for ET or (b) endocrine resistant pathways and phenotypes (epithelial, epithelial-mesenchymal transition, mesenchymal) (Table S1) and established and validated them on a single cellular level with endocrine responsive MCF-7, endocrine resistant MCF-7/TAMR1, and endocrine unresponsive MDA-MB-231 cell line cells. Single MCF-7, MCF-7/TAMR1, and MDA-MB-231 cells with an intact morphology and good RNA quality (i.e., Cq < 30 for *ACTB* and *KR19*) were used for in-depth multi-marker profiling (Figure 2A). In addition, 87% of measured transcripts could be consistently detected in single cell line cells as well as in pools of five cells. Unsupervised hierarchical clustering of gene expression profiles for two single cells as well as for pools of five cells of each MCF-7, MCF-7/TAMR1, and MDA-MB-231 cells revealed mostly comparable gene expression profiles for samples of the same cell line. Single MCF-7, MCF-7/TAMR1, and MDA-MB-231 cells are reproducibly clustered by cell line designation and demonstrated cell line specific characteristics. MCF-7 and MDA-MB-231 cells differed significantly with regard to expression of *EpCAM*, *KR19*, *VIM*, *CD24L4*, *ESR1*, *PGR*, and *IGFR* (Figure 2B). Thus, expression data confirmed the epithelial, endocrine-responsive phenotype of MCF-7 cells compared to the endocrine non-responsive, mesenchymal-like phenotype of MDA-MB-231 cells in single cells as well as pools of five cells. MCF-7 cells revealed a significantly higher expression of *PGR* compared to MCF-7/TAMR1 cells (Figure 2C).

### 2.2. Clinical Characteristics and Therapeutic Interventions of the DLA Patient

**Figure 3:**
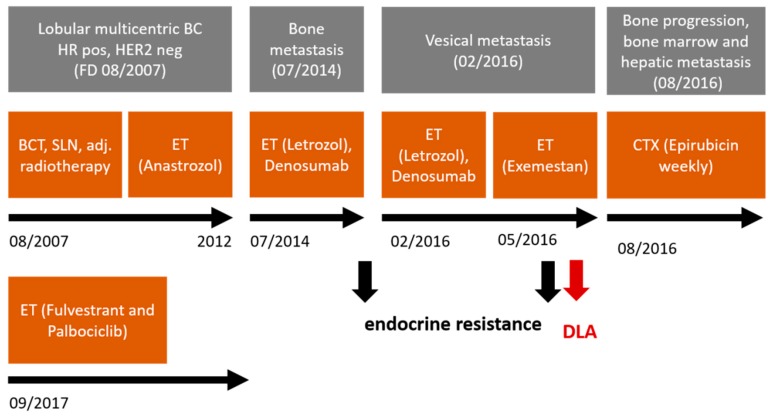
Clinical characteristics and therapeutic interventions. The time-point of performed Diagnostic Leukapheresis (DLA) is depicted by the red arrow. The black arrows mark the appearance of therapeutic endocrine resistance.

Clinical characteristics of the patient, who underwent DLA, and the therapeutic interventions administered to her are depicted in Figure 3. The Caucasian 65-year-old female patient was first diagnosed with a multi-centric right sided BC in 2007. Receptor status was positive for ER and PR (progesterone receptor) and negative for HER2 (human epidermal growth factor receptor 2). In the course of the disease, the patient developed bone, vesical, and hepatic metastatic lesions. DLA was performed when the patient suffered from a metastatic progress under ET with an aromatase inhibitor (AI).

### 2.3. Determination of Intra-Patient Heterogeneity by Multi-Marker Gene Expression Profiling of Uncultured and Cultured CTCs

#### 2.3.1. Uncultured CTCs

**Figure 4:**
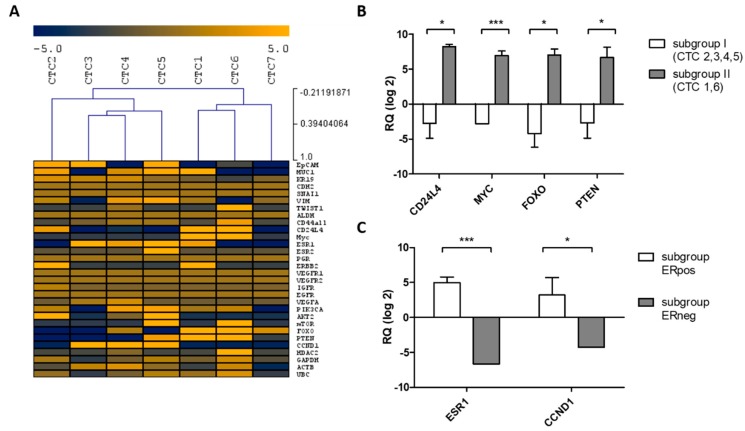
Gene expression of single CTCs. (**A**) Dendrogram and heat map analyses of Cq measurements of single CTCs (*n* = 7). Each gene is measured in triplicate. Data are mean centered, with mean expression responses to zero. Yellow indicates high expression and the blue color represents downregulation relative to the mean of the pool. (**B**) Bar graph showing significant differences in transcript expression between subgroup I (CTC 2,3,4,5) and subgroup II (CTC 1,6) (*, *p* < 0.05, *** *p* < 0.001). (**C**) Bar graph showing significant differences in transcript expression between the ER-positive CTC subgroup and the ER-negative CTC subgroup (* *p* < 0.05, *** *p* < 0.001).

Gene expression was measured in seven uncultured CTCs of intact morphology and good RNA quality after processing the cryopreserved DLA sample with the microfluidic Parsortix^TM^ and CellCelector^TM^ micromanipulation systems (Figure 4A). A total of 77% of the 30 evaluated genes were consistently detectable. Gene expression analysis of single CTCs revealed a high plasticity as well as intra-individual heterogeneity in terms of endocrine and phenotypic markers. Both ER-positive and ER-negative CTCs were present. Moreover, HER2-positive CTCs were detectable despite the HER2 negative primary tumor and growth factor receptors (EGFR, IGFR, and FGFR) were expressed in CTCs. Single CTCs also revealed an Epithelial-Mesenchymal Transition (EMT) marker (*TWIST*) as well as a transcript associated with a mesenchymal phenotype (*VIM*). Transcripts of the stem cell-associated marker *CD44* could be detected in all CTCs. Unsupervised hierarchical clustering indicated that there might be two clusters and stratified CTCs into two subgroups. CTC7 demonstrated substantially lower overall transcript levels than the other six CTCs. Additionally, expression of *ACTB* was reduced. For the other six CTCs, expression of reference genes showed a similar range of variability in both subgroups, but expression differed significantly with regard to phenotypic markers and markers of endocrine signaling pathways. In Subgroup I, the stem cell associated marker *CD24L4*, the proto-oncogene *MYC*, the transcription factor *FOXO*, and the tumor suppressor gene *PTEN* were expressed at significantly higher levels (Figure 4B). Importantly, a comparison of ER-positive versus ER-negative CTCs detected a significant higher expression of *CCND1* in the ER-positive CTCs compared to the ER-negative subgroup. *CCND1* is a key gene in regulating the cell cycle. ER regulated its expression (Figure 4C).

#### 2.3.2. Cultured CTCs

**Figure 5:**
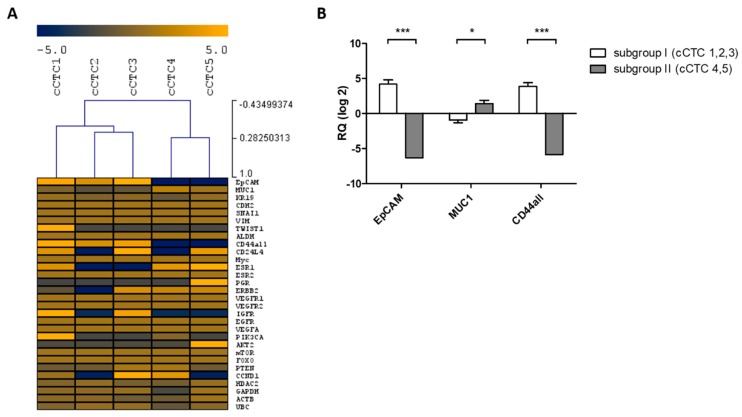
Gene expression of single cultured CTCs. (**A**) Dendrogram and heat map analyses of Cq measurements of single cCTCs (*n* = 5). Each gene is measured in triplicate. Data are mean centered, with mean expression responses to zero. Yellow indicates high expression and blue color represents downregulation relative to the mean of the pool. (**B**) Bar graph showing significant differences in transcript expression between subgroup I (cCTC 1, 2, 3) and subgroup II (cCTC 4, 5) (* *p* < 0.05, *** *p* < 0.001).

Gene expression profiling of single cCTCs of the cryopreserved DLA sample was performed after long-term culture for four weeks (Figure 5A). A total of 56% of measured genes could be detected and gene expression analysis revealed ER-positive as well as ER-negative cCTCs. Moreover, cCTCs were HER2-positive as well as HER2-negative. All cCTCs expressed *MUC1*. Unsupervised hierarchical clustering stratified cCTCs into two subgroups, which differed significantly in the expression of phenotypic markers (Figure 5B). Subgroup I showed a significantly higher expression of the epithelial marker EpCAM and the stem cell associated marker *CD44*. In contrast, subgroup II revealed a significantly higher expression level of the oncogene *MUC1*.

#### 2.3.3. Concordance between Profiles of Uncultured and Cultured CTCs

**Figure 6:**
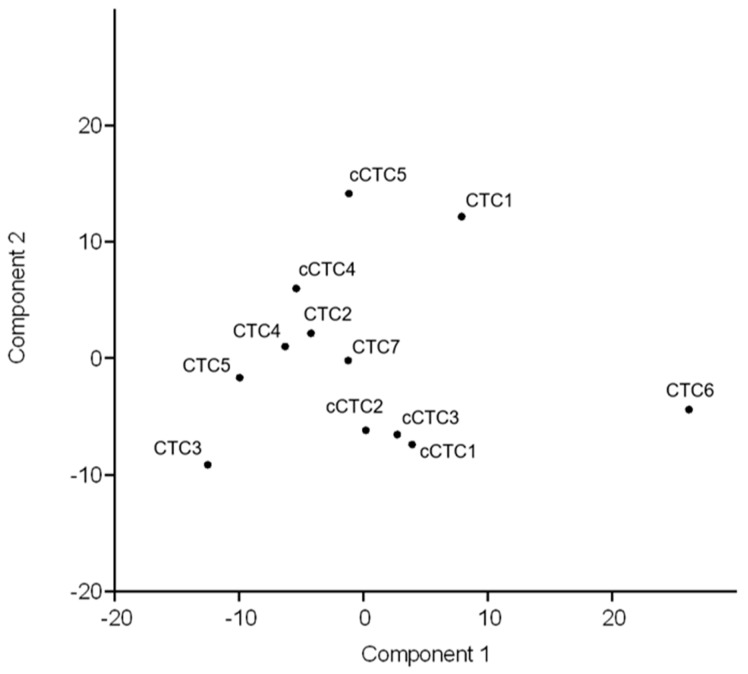
Combined gene expression analysis of single uncultured and cultured CTCs. PCA showed no clear distinction, but rather showed a partly combined grouping of CTCs (*n* = 7) and cCTCs (*n* = 5). Uncultured CTCs are distributed to a greater extent.

In an effort to evaluate the similarities between uncultured and cultured CTCs of the same patient, we combined single cell expression data in a principal component analysis (PCA) (Figure 6) and hierarchical clustering analysis (Figure S2). Inter-cellular heterogeneity of therapeutic relevant target genes (*ESR1, HER2*) could be observed in both uncultured CTCs and cCTCs. When all 30 genes were considered in the PCA, gene expression profiles of CTCs and cCTCs showed no clear distinction and showed a partly combined grouping. However, PCA revealed a higher intra-individual heterogeneity of uncultured CTCs in comparison to cultured CTCs visible by their wider distribution in the PCA plot.

## 3. Discussion

Tumor heterogeneity is a major challenge in effective endocrine BC treatment. Resistance to ETs is observed in up to 40% of BC patients [15]. Several molecular resistance mechanisms have been proposed besides tumor heterogeneity [3,4]. Breast cancers consisting of distinct tumor heterogeneity may be more likely to contain ET resistant cellular subclones. Real-time detection and monitoring of potential resistant subclones would be a crucial contribution to the choice of ET regimes. Respectively, characterization of CTCs and detection of molecular signatures in real-time is an important step toward targeted therapies and might, therefore, help to reveal valuable information concerning intra-patient heterogeneity relevant to therapeutic interventions.

Analysis of CTCs is currently challenged by their low numbers in peripheral blood. Our previous studies have shown that DLA provides higher numbers of CTCs per cancer patient and that it increases the likelihood of successful CTC culture [8,10]. Therefore, DLA may surmount the challenges of CTC detection in peripheral blood, which will be crucial for the successful implementation of molecular CTC analysis, especially concerning single cell diagnostics and culturing of CTCs. In addition, cryopreservation of DLA products allows long-term storage and easy distribution between different laboratories without affecting their morphology and quality [10]. One challenge to process aliquots of DLA-samples is their high content of white blood cells (WBCs), which is normally beyond the separation capacities of most CTC-isolation devices. The label-free microfluidic Parsortix^TM^ platform has been developed to separates CTCs by size and deformability while keeping CTCs viable [16] and has the ability to process blood volumes larger than 7.5 mL. We, therefore, selected this method to develop a robust, cost-effective workflow for enrichment of CTCs from DLA-products by combining Parsortix^TM^ with single cell micro-manipulation using the CellCelector^TM^. In our hands, it took five hours to the maximum to enrich and isolate single cells for transcriptome analysis. 

Single cell analysis depicts the true diversity of a heterogeneous cell population whereas a molecular analysis of CTC pools obscures potential CTC-subgroups, intra-patient heterogeneity, and the individual cell biology. Concerning performed RT-qPCR analysis, false positive rates due to the formation of primer dimers or other aberrant products could be avoided by the use of probe-based assays. Moreover, aberrant PCR products were minimized by assay optimization and by rejecting Cq values > 35 [17]. Normalization of expression data to reference genes was not performed with regard to single cell burst kinetics [18]. We found that expression analysis revealed high detection rates of targeted transcripts down to a single cellular level. Gorges et al. recommended the performance of additional “qualitative PCR” tests besides an intact cell morphology as quality control before single cell multi-marker analyses [14]. *KR19* has been widely used as a CTC indicator [12] as well as a quality marker of CTCs for further single cell analytics [14]. Respectively, the expression levels of *ACTB* and *KR19* served as a quality indicator to select cells and mRNA products for further multi-marker profiling. In order to analyze phenotypic CTC plasticity, we defined a Cq < 30 for *ACTB* and *KR19* as a threshold based on *KR19* and *ACTB* transcripts of single epithelial MCF-7 and mesenchymal MDA-MB-231 cells. A good mRNA quality-indicated by a Cq < 30 of *ACTB* and *KR19*-revealed 75% of single cell line cells, 30% of uncultured CTCs, and 50% of cCTCs. Moreover, CTCs (e.g., CTC 7) with overall low transcript levels of the analyzed genes might be damaged during processing and should be excluded from analysis [14].

According to previous studies, establishment and validation of the multi-marker gene expression analysis on a single cellular level was performed with cell line cells [14,19]. Gene expression data of single and pools of MCF-7, MCF-7/TAMR1, and MDA-MB-231 cells revealed mostly comparable gene expression profiles, reproducibly clustered by cell line designation and demonstrated cell line specific characteristics. Respectively, gene expression data displayed the epithelial, endocrine-responsive phenotype of MCF-7 cells and the endocrine non-responsive, mesenchymal-like phenotype of MDA-MB-231 cells. In line with Cremoux et al., profiling of MCF-7/TAMR1 cells revealed a significant lower expression of *PGR* compared to parental MCF-7 cells [20]. Concerning inter-cellular heterogeneity, variable gene expression of clonally identical cell line cells on a single cellular level might be due to transcriptional bursting: the naturally given stochastic activation and inactivation of promoters, which occurs in transcriptional pulses [21,22].

Single CTC analysis enabled us to display the inter-cellular heterogeneity as well as the identification of different CTC subgroups within one patient. CTCs as well as cCTCs showed a high epithelial/mesenchymal plasticity as well as intra-individual heterogeneity in terms of the expression of endocrine and phenotypic markers (*ESR1, PGR, HER2, IGFR, VEGFA, PIK3CA, AKT2, mTOR, FOXO, PTEN, CDK1, CCND1,* and *HDA*C2). In an analogy to previous reports, inter-cellular heterogeneity of relevant ET markers as well as markers associated with endocrine resistance (e.g., *ESR1, HER2, PI3KCA, FGFR1,* and *CCND1*) could be observed [23]. Single CTCs and cCTCs demonstrated a high *HER2* expression whereas the primary tumor was negative for *HER2*. Discordances in the HER2 status between CTCs and corresponding primary tumors were reported in previous studies [24,25,26]. 

Focusing on the single expression data of CTCs and cCTCs, different subgroups were identified. These were characterized by a significant different expression of phenotypic markers (*EpCAM, CD44, CD24, MYC,* and *MUC1*) and markers of endocrine signaling pathways (*FOXO* and *PTEN*). Dysregulation of *FOXO* and *PTEN* is associated with endocrine resistance [27,28]. In addition, *EpCAM*, *CD44*, and *CD24* have been reported to be relevant for tumor progression and metastatic processes [29,30].

The comparison of the ER-positive and the ER-negative CTC subgroup revealed a significant higher expression of Cyclin D1 (*CCND1*) in the ER-positive subgroup. The CyclinD1/CDK4/CDK6 pathway is a key regulator of the cell cycle. Increased CyclinD1 expression is associated with proliferation and endocrine resistance in ER-positive BCs [31]. CDK4/6 inhibitors are a new standard-of-care therapy in ER-positive metastatic BC. Combinations of CDK4/6 inhibitors with AIs or SERDs/SERMs (selective ER down regulators/selective ER modulators) significantly improved progression free-survival of patients with ER-positive metastatic BC [32,33,34]. 

All CTCs expressed *CD44* whereas all cCTCs expressed *MUC1*. *CD44* and *MUC1* have been reported to be relevant for proliferation, invasion, apoptosis, metastatic processes and therapy resistance [35,36]. However, expression of *MUC1* in cCTCs might be influenced by the cell culture media, especially by the supplement of hEGF and its interactions with *EGFR* and *MUC1* [37].

In vitro culture of CTCs is a rational strategy for large scale propagation of the limited CTC numbers present in patient samples, to derive enough CTCs to test functional responses to putative druggable targets based on their biology and expression patterns. To date, there are no published studies of single cell expression data concerning uncultured CTCs and cCTCs of the same BC patient, which may be caused by the fact that all CTCs of a blood sample are used for the in vitro culture. In our approach, DLA enables direct comparison of patient-matched CTCs and cCTCs, which is important to test the idea to use cCTCs for potential drug screening. DLA-aliquots will also enable the comparison of CTCs to their pendants cultured in animal models. In this case, PCA displayed no clear distinction, but rather shows a partly combined grouping of CTCs and cCTCs. Higher intra-individual heterogeneity of uncultured CTCs was observed in comparison to cCTCs. Intra-individual heterogeneity of cCTCs as well as expression levels of stem cell markers and the Tumor Sphere Medium and growth stimulating supplements may influence its downstream signaling pathways. Moreover, inter-cellular heterogeneity of therapeutic relevant target genes (*ESR1, HER2*) was seen in CTCs as well as cCTCs. This may support the idea of using cCTCs for pharmacologic testing even though more DLA products obtained from more BC patients have to be investigated.

The analyzed metastatic BC patient revealed an AI resistance at the time of the DLA procedure. AI resistance was reported to be associated with increased tumor heterogeneity, loss of ER expression, *ESR1* mutations, or upregulation of growth factors [38,39]. These possible endocrine resistance mechanisms could be partly displayed by the shown single cell expression data of CTCs and cCTCs. ER-positive and ER-negative CTCs could be detected after AI treatment. Reasons for increased CyclinD1 expression in the ER-positive CTC subgroup, despite anti-estrogen therapy with an AI treatment, might be because of activating *ESR1* mutations. Persistence of ER-negative CTCs under AI therapy might be due to mechanisms, which could not be detected by the chosen multi-marker panel. Endocrine-based therapies with CDK4/6 inhibitors can overcome AI resistance. Therefore, the patient was treated according to clinical practice guidelines with fulvestrant and palbociclib. Concerning the therapy response of CDK4/6 inhibitors, Cyclin D1 expression or *CCND1* amplification might play a relevant role [40]. Further CTC analyses under CDK 4/6 inhibitor therapy might give insight into responding subpopulations.

## 4. Materials and Methods 

### 4.1. Cell Culture

MCF-7 and MDA-MB-231 cells were obtained from the American Type Culture Collection (Manassas, VA, USA). MCF-7/TAMR1 cells were purchased from Merck (Darmstadt, Germany). MCF-7 and MDA-MB-231 cells were maintained in DMEM/F12 medium without phenol red, containing 1% fetal bovine serum, 2.5 mM L-Glutamine, 6 ng/mL insulin, and 100 units/mL penicillin-streptomycin in a humidified incubator at 37 °C with 5% CO_2_. Respectively, 1 µM tamoxifen was supplemented in MCF-7/TAMR1 cells. Cultured cells were harvested at a confluence of approximately 80%.

### 4.2. Diagnostic Leukapheresis 

Diagnostic Leukapheresis (DLA) was performed at the Department of Transplantation Diagnostics and Cell Therapeutics, Duesseldorf, Germany, as previously described [7,9]. Aliquots of the DLA sample were frozen and stored for future use. Cryopreservation of DLA samples was described by our group before [10].

### 4.3. Enrichment of Viable CTCs from Cryopreserved DLA Samples

Cryopreserved DLA samples (10^8^ mononuclear cells) were rapidly thawed in a 37 °C water bath and filtered through a fine sieve (mesh size 100 µm). The filtered DLA sample was diluted as 1:20 in PBS. Subsequently, viable CTCs were enriched with the Parsortix™ system (Angle plc, Guildford, UK). The Parsortix™ system was used, according to the manufacturer’s instructions. Following the protocol, filtration cassettes with 6.5 µm gaps were used and a 100 mbar of pressure was applied [10,16]. 

### 4.4. CTC Culture

For the CTC culture, Tumor Sphere Medium was used as previously described [10,41]. CTCs were maintained in RPMI 1640 Medium supplemented with 20 ng/mL human epidermal growth factor, 1× B27, 20 ng/mL fibroblast growth factor and 1% penicillin-streptomycin in a humidified incubator at 37 °C with 5% CO_2_ and 4% O_2_.

### 4.5. Staining and Isolation of Single Cells

Cell line cells were harvested, washed, and transferred onto a glass slide for single cell isolation. CTCs were stained in parallel for Hoechst, EpCAM (VU1D9, Stemcell^TM^ Technologies, Vancouver, BC, Canada) and CD45 (3S-Z5, Santa-Cruz Biotechnology, Dallas, TX, USA) for identification. Stained cells were washed with PBS and transferred onto a glass slide for single cell isolation by micromanipulation. Micromanipulation of single cells by the CellCelector^TM^ system (ALS, Jena, Germany) was previously described [42].

### 4.6. RNA Isolation, cDNA, and RT-qPCR

RNA from single cells or cell pools was isolated using ProteinaseK (pre-digestion, 2 mg/mL) and the CelluLyser™ Micro Lysis Kit (TATAA Biocenter, Göteburg, Sweden). RNA was reversely transcribed using the GrandScript cDNA Synthesis Kit (TATAA Biocenter, Göteburg, Sweden). Subsequently, pre-amplification was done with the GrandPerformance Cancer Panel PreAmp Primer Mix^®^ and the PreAmp Grand Master Mix^®^ (TATAA Biocenter, Göteburg, Sweden). Pre-amplification was performed as follows: pre-denaturation at 95 °C for 60 s, followed by 17 cycles of 95 °C for 15 s, 60 °C for 2 min, and 72 °C for 1 min. RT-qPCR was performed according to the manufacturer’s instructions using the LightCycler^®^ 480 II system (Roche, Basel, Switzerland). PCR was performed as follows: pre-denaturation at 95 °C for 60 s, followed by 40 cycles of 95 °C for 5 s, and 60 °C for 1 min. All kits were applied according to the instructions of the vendors. 

### 4.7. Statistical Analysis and Single Cell Analysis

Data are expressed as mean ± SEM. Statistical analyses were performed by ANOVA followed by Bonferroni’s test or Student’s *t*-test using the Prism analysis program (GraphPad Inc., San Diego, CA, USA). *p* < 0.05 was considered to be statistically significant. Single qPCR data analysis was performed as previously described [14,17]. A quantification cycle (Cq) value of 37 replaced missing data values. Cq values greater than 35 were treated as off-scale data points. Next, Cq values were converted to relative quantities of cDNA molecules (RQ) and transformed to log2-scale. Due to the stochasticity of single cell expression patterns, data was not normalized to reference genes. Respectively, single cell expression levels were displayed as relative quantities per cell. Multivariate qPCR expression profiling was done by performing hierarchical clustering and principal component analysis (PCA). Respectively, expression data was mean-centered.

### 4.8. Patient Samples

Analysis of human samples was carried out in accordance with Good Clinical Practice guidelines and was approved by the Ethics Committee of the Medical Faculty of the Heinrich-Heine-University Duesseldorf (Ref-No: 3460). Written informed consent was obtained from the patients. BC samples were obtained from the Department of Obstetrics and Gynecology, University Hospital and Medical Faculty of the Heinrich-Heine-University Duesseldorf, Germany.

## 5. Conclusions

A robust, rapid, and cost-efficient workflow was successfully established for enrichment of single CTCs from DLA aliquots using the microfluidic Parsortix^TM^ system combined with CellCelector^TM^ micromanipulation. This workflow enables subsequent multi-marker transcriptomic profiling for endocrine resistance mechanisms as well as relevant therapeutic targets for ETs. For the first time, single gene expression profiles of uncultured and cultured CTCs of the same metastatic BC patient could be generated. Although the number of CTCs and cCTCs studied in this proof-of-principle study was small, gene expression profiles suggested possible endocrine resistance mechanisms as well as relevant therapeutic targets for ETs. Lastly, these technologies constitute promising tools for CTC analysis, which might have great potential for clinical decision-making in the future, especially if tissue biopsies might not be available. However, standardization and optimization of protocols as well as translational studies are necessary.

## Supplementary Materials

The following are available online at https://www.mdpi.com/2072-6694/11/7/903/s1, Table S1: Gene panel, Figure S1: Single cell analytics, Figure S2: Combined gene expression analysis of single uncultured and cultured CTCs.
