# Supplementary material for: Diagnostic Leukapheresis Enables Reliable Transcriptomic Profiling of Single Circulating Tumor Cells to Characterize Inter-Cellular Heterogeneity in Terms of Endocrine Resistance
Source: Cancers (Basel). 2019 Jun 28;11(7):903. doi: 10.3390/cancers11070903 (PMC6679140; doi:10.3390/cancers11070903)
Supplement: Supplementary file 1 [file cancers-11-00903-s001.pdf]

Supplementary Materials: Diagnostic Leukapheresis Enables Reliable Transcriptomic Profiling of Single Circulating Tumor Cells to Characterize Inter-Cellular Heterogeneity in Terms of Endocrine Resistance

Florian Reinhardt, André Franken, Franziska Meier-Stiegen, Christiane Driemel, Nikolas H. Stoecklein, Johannes C. Fischer, Dieter Niederacher, Eugen Ruckhaeberle, Tanja Fehm and Hans Neubauer

Table S1. Gene panel. Genes used to profile single cell line cells, cCTCs and CTCs.

EpCAM	Epithelial Cell Adhesion Molecule
MUC1	Mucin 1
KRT19	Keratin 19
CDH2	Cadherin 2
SNAI1	Snail family zinc finger 1
VIM	Vimentin
Twist1	Twist family HHLH transcription factor 1
ALDH	Aldehyde dehydrogenase 1 family, member A1
CD_44 all	CD44 molecule
CD24L4	CD24 molecule
Myc	V-myc avian myelocytomatosis viral oncogene homolog
ESR1	Estrogen receptor 1
ESR2	Estrogen receptor 2
PGR	Progesterone receptor
ERBB2	V-erb-b2 erythroblastic leukemia viral oncogene homolog 2
EGFR	Epidermal growth factor receptor
VEGFR1	Fms-related tyrosine kinase 1
VEGFR2	Kinase insert domain receptor
IGFR	Insulin-like growth factor 1 receptor
VEGFA	Vascular endothelial growth factor A
PI3K	Phosphoinositide-3-kinase
AKT2	V-akt murine thymoma viral oncogene homolog 2
mTOR	Mechanistic target of rapamycin
FOXO	Forkhead box O3
PTEN	Phosphatase and tensin homolog
CCND1	Cyclin D1
HDAC2	Histone deacetylase 2
GAPDH	Glyceraldehyde-3-phosphate dehydrogenase
ACTB	Actin beta
UBC	Ubiquitin C

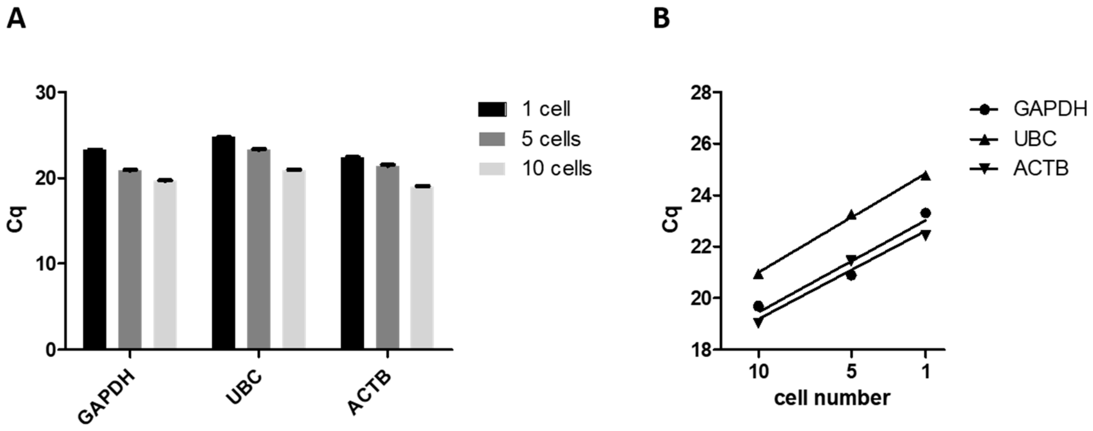

Figure S1. Single cell analytics (**A**) Cq measurements of the reference genes *GAPDH*, *UBC* and *ACTB* of 1, 5 and 10 MDA-MB-231 cells. Each gene is measured in triplicate. (**B**) Regression analysis of detected Cq measurements of the reference genes *GAPDH*, *UBC* and *ACTB* of 1, 5 and 10 MDA-MB-231 cells.

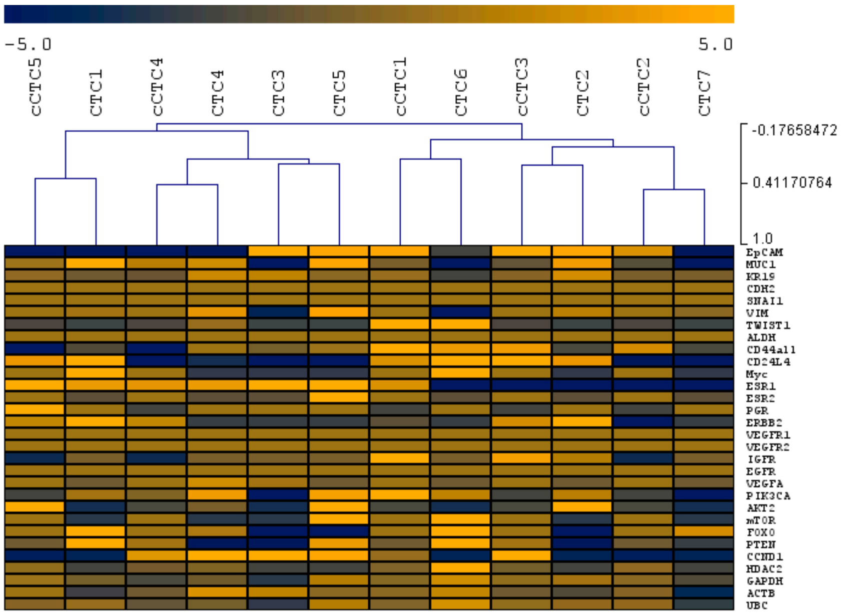

Figure S2. Combined gene expression analysis of single uncultured and cultured CTCs. Dendrogram and heat map analyses of Cq measurements of single CTCs (n = 7) and cCTCs (n = 5). Each gene is measured in triplicate. Data are mean centered, with mean expression responses to zero. Yellow indicates high expression and blue color represents downregulation relative to the mean of the pool.

© 2019 by the authors. Licensee MDPI, Basel, Switzerland. This article is an open access article distributed under the terms and conditions of the Creative Commons Attribution (CC BY) license (http://creativecommons.org/licenses/by/4.0/).
